# A Method for Detecting the Randomness of Barkhausen Noise in a Material Fatigue Test Using Sensitivity and Uncertainty Analysis

**DOI:** 10.3390/s20185383

**Published:** 2020-09-20

**Authors:** Yuting Hou, Xiang Li, Yang Zheng, Jinjie Zhou, Jidong Tan, Xiaoping Chen

**Affiliations:** 1 School of Aeronautics and Astronautics, University of Electronic Science and Technology of China, Chengdu 611731, China; 201821100223@std.uestc.edu.cn (Y.H.); xpchen@uestc.edu.cn (X.C.); 2 China Special Equipment Inspection and Research Institute, Beijing 100029, China; zhengyang@csei.org.cn (Y.Z.); tanjidong@csei.org.cn (J.T.); 3 School of Mechanical Engineering, North University of China, Taiyuan 030051, China; S1902036@st.nuc.edu.cn

**Keywords:** fatigue prediction, magnetic Barkhausen noise signal, uncertainty and sensitivity modeling, feature reparameterization

## Abstract

The magnetic Barkhausen noise (MBN) signal provides interesting clues about the evolution of microstructure of the magnetic material (internal stresses, level of degradation, etc.). This makes it widely used in non-destructive evaluation of ferromagnetic materials. Although researchers have made great effort to explore the intrinsic random characteristics and stable features of MBN signals, they have failed to provide a deterministic definition of the stochastic quality of the MBN signals. Because many features are not reproducible, there is no quantitative description for the stochastic nature of MBN, and no uniform standards to evaluate performance of features. We aim to make further study on the stochastic characteristics of MBN signal and transform it into the quantification of signal uncertainty and sensitivity, to solve the above problems for fatigue state prediction. In the case of parameter uncertainty in the prediction model, a prior approximation method was proposed. Thus, there are two distinct sources of uncertainty: feature(observation) uncertainty and model uncertainty were discussed. We define feature uncertainty from the perspective of a probability distribution using a confidence interval sensitivity analysis, and uniformly quantize and re-parameterize the feature matrix from the feature probability distribution space. We also incorporate informed priors into the estimation process by optimizing the Kullback–Leibler divergence between prior and posterior distribution, approximating the prior to the posterior. Thus, in an insufficient data situation, informed priors can improve prediction accuracy. Experiments prove that our proposed confidence interval sensitivity analysis to capture feature uncertainty has the potential to determine the instability in MBN signals quantitatively and reduce the dispersion of features, so that all features can produce positive additive effects. The false prediction rate can be reduced to almost 0. The proposed priors can not only measure model parameter uncertainties but also show superior performance similar to that of maximum likelihood estimation (MLE). The results also show that improvements in parameter uncertainties cannot be directly propagated to improve prediction uncertainties.

## 1. Introduction

The Barkhausen effect specifically refers to the phenomenon in which the magnetic flux of a magnetized material changes discontinuously due to magnetic domain inversion during magnetization [1]. As an important nondestructive testing (NDT) method, the magnetic Barkhausen noise (MBN) signal is sensitive to the changes in many material properties and has many applications in the fields of material stress and hardness detection [2,3,4,5], metal fatigue state analysis [6], metal microstructure transformation, and grain size measurement [7].

As the direct carrier for excitation and reception of MBN signal, the characteristics of sensor determine the quality of the MBN detection result. We adopted a self-developed MBN sensor with high spatial resolution. In detail, the MBN sensor is mainly composed of an excitation device and an MBN signal receiving device. The excitation part is a U-shaped yoke composed of a plurality of overlapping silicon steel sheets, and the excitation coil is composed of a multi-hit coil wound around the U-shaped yoke, which are used to excite an alternating magnetic field. The receiving device uses a magnetic core-wound coil receiver, which is generally placed between the two yokes of the U-shaped yoke while keeping perpendicular to the test piece for measurement. In order to improve the spatial resolution and measurement stability of the sensor, we add a layer shielding shell with high permeability and conductivity to the outside of the receiver, limiting the effective signal receiving area of the MBN sensor to a relatively small local area. In addition, we increased the number of turns for the pickup coil, so as to achieve the purpose of getting good sensitivity and frequency response characteristics in a lower frequency range.

**Figure 1:**
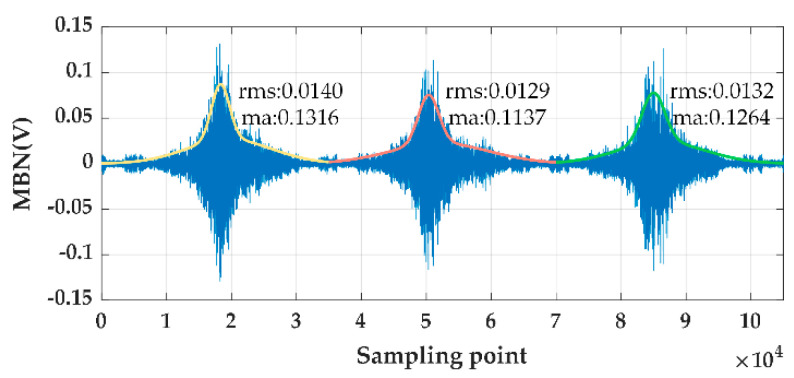
Random characteristics of magnetic Barkhausen noise (MBN) signal.

Intensity changes in an MBN signal with material state transformation are usually reflected and described by the representative parameters calculated in time-frequency (TF) domain such as the amplitude, energy, root mean square (RMS), waveform full width at half maximum (FWHM), envelope, peak time, threshold, and power spectrum [3,4,5,8,9,10]. However, affected by the microscopic magnetic anisotropy of the material itself, measurement performance, and experimental magnetization parameters (such as magnetization intensity and frequency, excitation waveform), the MBN has an obvious stochastic nature and the application of more automatic signals processing procedures used for extraction, selection, and fusion of signal features containing critical and distinctive information about the material properties are urgently required. Figure 1 provides an intuitive understanding of the stochastic characteristic of the MBN signal, in which distinctive differences can be drawn from different wave packets of MBN signals. According to the relevant literatures, multiple signal processing methods have been applied to get more representative information of MBN signal. Magalas [11] introduced wavelet transform into MBN signal analysis; then, Miesawicz et al. [12] further expanded the study. Luo et al. [13] applied the auto-regressive modeling method to obtain a single-sided power spectrum (also called a PSD or an AR spectrum), which is a near-deterministic expression of the MBN signal. In [14,15,16], Hilbert–Huang Transform and Short-Time Fourier Transform (STFT) are used to analyze the joint TF representation and properties of MBN signal. In addition, wide new features are also proposed. Vashista et al. [17] proposed two parameters of “count” and “event” of MBN signal; on this basis, Hang [10] used a genetic algorithm (GA) for appropriate threshold selection. Su et al. [4] performed first- and second-order derivation on the AR spectrum and manually extracted some peaks from it as new features. Li et al. [5] adopted a modified slow feature analysis (m-SFA) to enrich the content of PSD by fusing the AR spectrum with different orders. There are many types of features, which can be roughly divided into energy feature, statistical feature, and shape feature, and great differences may manifest for different features used in different applications. When a variety of new features continue to be extracted, the problem of feature explosion arises, and various dimensionality reduction methods are needed to decrease the redundancy of features such as PCA [4,9], FCA [9], and forward-selection combined with cross-validation [18]. It is complex work.

Although researchers have proposed many excellent methods for MBN signal processing so that features can have good linearity and low dispersion, reducing the influence of the random characteristics of MBN signal on the detection or prediction results. There are three problems that must be considered in these methods. The first one is that many new features are not reproducible on cycling. In some cases, once the materials, experimental equipment and methods are changed, the new features may be useless and are not always superior to the conventional features, lacking in universality and versatility. The second, definite explanations and definitions about the laws of the stochastic nature of MBN signal haven’t been made. Thirdly, due to the particularity of different features, we cannot use uniform standards to analyze, define, and classify the performance of features, making the features analysis and selection complicated.

Summarizing the works of others, we aim to make further study on the internal quality of the random characteristics of MBN signal and transform it into the accurate quantification of signal uncertainty and sensitivity, to solve the three problems above. For data uncertainty measurement, the most commonly used are probabilistic methods based on data statistics such as Monte Carlo method and Bayesian analysis [19]. Among them, Bayesian analysis has a great advantage in making full use of historical information and continuously integrating new data to learn the accurate probability distribution of data when the sample is unbalanced and short. In Bayesian learning, clarifying the role of prior and likelihood information in Bayesian inference of large models is necessary. Likelihood reflects the amount of data to some degree and Prior represents how certain the researcher is about model parameters being estimated [20]. In [21,22], the discussion has also been given out. It can be concluded that, when accurate informed priors are specified, the amount of theory (via the prior) incorporated into the model estimation process increases. Specifically, with strong and accurate priors, less data would likely be needed to properly estimate parameter values. In contrast, more data would be needed in cases where no theory was incorporated. Helton et al. [23] carried out eigenvalue uncertainty analysis on nuclear data using a survey of sampling-based statistical methods and discussed the influence of data uncertainty caused by the data amount and the degree of dispersion on the uncertainty of model prediction. In [21], Sarah et al. used Bayesian statistics for mixture uncertainty modeling, illustrating the importance of a prior sensitivity analysis, and discussing how to interpret results that fluctuate with different prior settings.

Based on these analyses, in a complex system, two distinct sources of uncertainty can be considered: feature(observation) uncertainty and model uncertainty [24,25]. In this paper, we specify that the stochastic quality of MBN signal is characterized as features uncertainty. Model uncertainty arises from the inability to specify an exact value for a parameter that is assumed to have a constant value in the respective investigation, and accounting for uncertainty in model parameter estimations makes the model robust to alleviate overfitting without a need for regularization [26]. Specifically, Bayes by Backprop modeled on variational inference was proposed to measure the weight uncertainty in a neural network [27]. Likewise, we try to model the parameters uncertainty of a multivariable linear regression (MLR) model, in which the posterior distribution of parameters can be inferred by Bayes’ theorem without using complicated variational inference. Moreover, output uncertainty analysis can be used to study how the sensitivity propagates from the uncertainty changes of input features and model parameters to the model predictions, which is called the prediction uncertainty. In other words, uncertain outputs can be treated as functions of uncertainty analysis of inputs and model parameters. In general, we aim to quantify uncertainty in the input features of MBN signals, uncertainty in the parameters of the learning model, and uncertainty in the output predictions produced by the input and parameters characterized as probability distribution based on the modeling of sensitivity and uncertainty. This has the potential to improve modeling flexibility and accuracy. The benefit of using Bayesian learning in our study is that they offer a unified and consistent set of tools for uncertainty and sensitivity measurements for modeling, inference, prediction, and model selection [28,29]. The methods given in this article include the following:

First, feature uncertainty is modeled by reparameterization sampling. Specifically, we perform interval sensitivity analysis based on the confidence intervals of the feature distribution. A certain interval value is selected as the measurement index of signal uncertainty by applying reparameterization sampling of feature matrices. When the uncertainty is maintained, a more robust feature group is reconstructed by uniformly quantizing the features extracted from the same signal wave packet. Then, model uncertainty is measured by BLR. For BLR, prior distribution (prior) selection and training are of great importance. Incorporating informed priors into the estimation process can improve convergence and prediction accuracy. Due to a lack of prior knowledge, we propose an effective method in this paper to assign model parameters more reasonable priors by calculating the gradient descent of the Kullback–Leibler (KL) divergence between the prior distribution and posterior distribution. In addition, prediction uncertainty is characterized as the statistical mean and variance of prediction for each fatigue state and is used as the evaluation index of the results of interval sensitivity analysis and prior sensitivity analysis. Prediction uncertainty is applied to measure the sensitivity of observation uncertainty and model uncertainty.

The rest of this paper is arranged as follows: Section 2 introduces the experimental setup and measurements. The main algorithm derivation and modeling process are illustrated in Section 3. Section 4 presents the experiment results and related analysis. Finally, the research in this paper is summarized and concluded in Section 5.

## 2. Experimental Setup and Measurements

The experimental system mainly consists of a low-frequency fatigue test machine (Shimadzu (China) Co., Ltd. Beijing, China) the MBN measurement instrument and the fatigue test piece. The measurement system was self-built and included a signal generator Handyscope hs3 (TiePie engineering, Amazing Tech Co., Ltd, Shenzhen, China), power amplifier Newton LPA05A (Newtons4th Ltd, Beijing Miko-Xinye Electronics Technology Co., Ltd. Beijing, China), preamplifier Stanford SR560 (Stanford Research Systems), self-developed current collector and magnetic Barkhausen signal sensor, and computer. The testing specimens are manufactured from carburized 20R steel (American: AISI/SAE, 1020, Britain: BS, IC22), whose chemical composition is shown in Table 1, and the full-size and shape were designed according to National standard. In the process of specimen preparation, the surface is ground to Ra1.6 roughness, then annealed at a high temperature of 550 °C for 6 h, and then the surface oxide scale with diluted hydrochloric acid is removed. The maximum magnetic field strength in the test sample is 99 A/m, and the saturation induction intensity of the sample is 1.5–2T, and the magnetic field intensity must be greater than 10,000 A/m when it reaches saturation. Then, tensile tests were carried out on specimens before the fatigue test. The lower yield strengths of the two specimens were 297 MPa and 301 MPa, and the corresponding tensile strengths were 443 MPa and 448 MPa. Through trial and error, 345 MPa was selected as the maximum tensile stress for the fatigue test. The test stress ratio was 0, and the stress change was in the form of a sinusoid wave with a frequency of 15 Hz. The MBN signal and the magnetic characteristic signal excited at the center of the specimen were measured after a certain cycle period.

**Figure 2:**
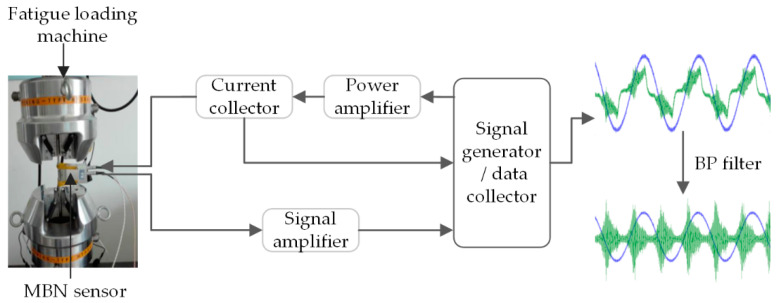
Experiment system.

The sensor used in the experiment is a magnetic core wound coil type shielded sensor designed based on non-destructive testing technology, which aims to solve the problem of low spatial resolution of the current coil wound sensor. The coil is made of manganese-zinc ferrite, and the noise of the coil itself without the specimen is consistent with the noise level of the system without the sensor. In the process of measurement, we use a rubber band to fix the sensor probe on the surface of the material. The tension of the rubber band is very small and can be ignored. The yoke of sensor contacts the specimen, and the pick-up is located between the two yokes and not in contact with specimen, so it is not affected by head pressure. The configuration block diagram of the experimental system is shown in Figure 2; during the experiment, the software on the computer controls the signal generator to emit a sinusoidal signal at the specified frequency. The sinusoidal signal is amplified by the power amplifier and then sent to the excitation coil through the current collector so that the alternating magnetic field is generated in the tested component and the MBN signal is excited.

Moreover, considering that the received signals are susceptible to the excitation signal and external factors, the measured MBN signals were filtered by a bandpass filter to remove the low-frequency and high-frequency interference signals. The detailed excitation and acquisition parameter settings are listed in Table 2. In order to better observe and compare the random characteristics of the different wave packets, we set a long signal acquisition channel to ensure that a sufficient number of signal wave packets (150) with the same sampling points (35,000) could be split from the continuous signal. Meanwhile, a dataset with enough samples was created for prediction.

The whole life cycle of ferromagnetic materials consists of the loading time from loading free to break. Due to the dispersiveness of fatigue, in order to reduce the experimental error, the same fatigue experiment and MBN signal measurement were carried out on three specimens taken from the same base material. The fatigue life cycle of three specimens measured in the experiment were 42,712 times, 54,643 times and 47,762 times, respectively. To explore the MBN signal across the whole life cycle of the ferromagnetic materials, the MBN signal was collected every 1000 cycles until the materials broke. To avoid the influence of experimental measurement errors, we randomly selected MBN signals under 12 measuring moments that corresponded to certain fatigue loading times for analysis, and we regarded each loading time as a different fatigue state. 

## 3. Modeling for the Uncertainty

Given a dataset, the choice of model and parametrization of the regression function are of the utmost importance. Our aim is to establish an accurate model using probability uncertainty and sensitivity analysis that does this reasonably well. This paper focuses on uncertainty modeling, including the uncertainty analysis of features, model parameters, and predictions. In any practical setting, we have access to only a finite, potentially large, amount of data for selecting the model class and the corresponding parameters. Given that this finite amount of training data does not cover all possible scenarios, we may want to describe the remaining parameter uncertainty to obtain a measure of confidence of the model’s prediction at test time. The smaller the training set is, the more important the uncertainty modeling.

**Figure 3:**
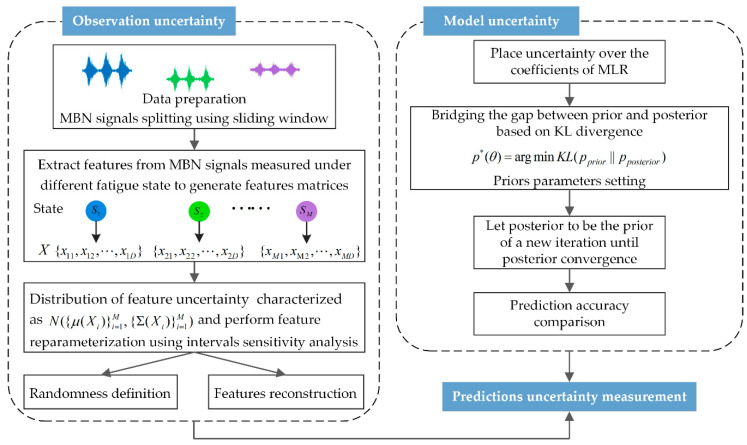
Flowchart of uncertainty modeling.

Consistent modeling of uncertainty provides model predictions with confidence bounds. The overall implementation process of this paper is illustrated in Figure 3 and includes two main parts. The first part summarizes the modeling process of observation uncertainty, in which signal preprocessing and feature extraction are carried out and the uncertainties in the signal are characterized as multiple normal distributions of the features. The second part is the measurement of model parameter uncertainties for prior setting and posterior training.

Given a set of training example pairs $D=\{(x_{1},y_{1}),(x_{2},y_{2}),\cdots ,(x_{N},y_{N})\}$, we aim to find a function f that maps input $x\in R^{K}$ to corresponding function values y∈R. For this dataset, N and K denote the size of the feature matrices characterized as $X=\{x_{1},x_{2},\cdots ,x_{N}\}\in R^{N\times K}$. Each element in the sample space X refers to an example with K features. We index the examples and features as n=1,2,⋯,N and k=1,2,⋯,K, respectively. We assume that a linear model is used for prediction. The functional relationship between x and y is given by

(1) $$y=x^{T}\theta +\varepsilon ,\;\varepsilon \sim N(0,\sigma^{2})$$

where $\theta \in R^{K}$ are the model parameters (regression coefficients) that we are about to measure and ε is an independent random variable that describes the measurement noise, which can also be called zero-mean Gaussian-distributed noise.

### 3.1. Observation Uncertainty Analysis Based on Reparameterization Sampling

In this section, the method of modeling observation uncertainty using probability distributions is given. The stochastic nature of MBN signal in this paper can be understood as signal instability, which affects the robustness and characterization of features. That is, the same feature extracted from different wave packets has different values. Based on these considerations, we aim to reconstruct the features to varying reparameterization degrees (manifested as confidence intervals of a Gaussian distribution) from the probability perspective to infer and define the uncertainty in the MBN signals. 

**Figure 4:**
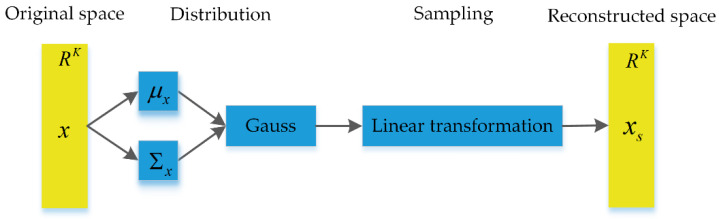
Feature reparameterization.

In observation uncertainty analysis of any multi-to-one response system using sampling methods, three steps are mainly considered: (1) Calculate the mean vector and covariance matrices to define the probability distribution for observation uncertainty characterization; (2) Reconstruct the sample space of observations by reparameterization sampling; and (3) Send the reconstructed sample space into the predictor for the response, in addition to analyzing the uncertainty in the response. The feature transformation based on reparameterization sampling is shown in Figure 4.

Feature reparameterization is conducted based on the substantial wave packets of the same fatigue states. To reconstruct the sample space, the distribution space and joint probability density function of each input parameter $x\in R^{K}$ must be determined first. Let a multivariate Gaussian distribution approximate the spatial probability distribution of the input parameters, which is parameterized by a mean vector $\mu_{x}\in R^{K}$ and a covariance matrix $\Sigma_{x}$. We write $X\sim N(\mu_{x},\Sigma_{x})$, and the joint probability density is given as

(2) $$p(x|\mu_{x},\Sigma_{x})={\left(2\pi \right)}^{-\frac{K}{2}}|\Sigma {|}^{-\frac{1}{2}}\exp(-\frac{1}{2}{\left(x-\mu_{x}\right)}^{T}|\Sigma_{x}{|}^{-1}(x-\mu_{x}))$$

The empirical mean vector $\mu (\mu =[\mu_{1},\mu_{2},\cdots ,\mu_{K}])$ and covariance matrix are defined as

(3) $$\mu_{x}=\frac{1}{N}\sum_{n=1}^{N}x_{n}$$

(4) $$\Sigma_{x}=Cov[x,x]=\left[\begin{array}{cccc} \mathrm{cov}[x_{1},x_{1}] & \mathrm{cov}[x_{1},x_{2}] & \cdots & \mathrm{cov}[x_{1},x_{K}]\\ \mathrm{cov}[x_{2},x_{1}] & \mathrm{cov}[x_{2},x_{2}] & \cdots & \mathrm{cov}[x_{2},x_{K}]\\ \vdots & \vdots & \ddots & \vdots \\ \mathrm{cov}[x_{K},x_{1}] & \cdots & \cdots & \mathrm{cov}[x_{K},x_{K}] \end{array}\right]$$

where $\mathrm{cov}[x_{i},x_{j}]$ is the covariance between two univariate random feature variables. The covariance matrix is symmetric and positive semidefinite and tells us something about the spread of the data space. The elements on its diagonal represent the variance of the features. 

**Figure 5:**
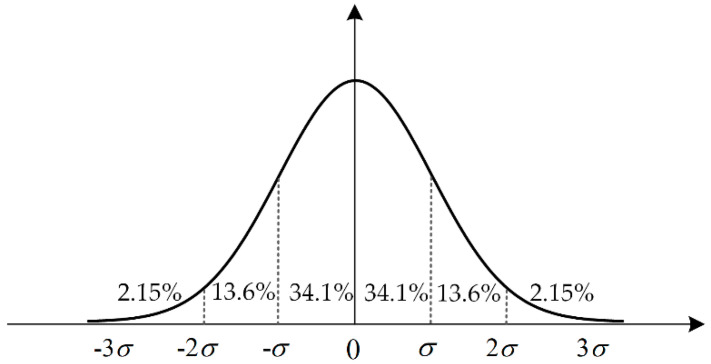
Selection of confidence intervals for standard normal distribution.

For a general multivariate Gaussian distribution, that is, where the mean is nonzero and the covariance is not the identity matrix, characterized as $N(\mu_{x},\Sigma_{x})$, we use the properties of linear transformation of a Gaussian random variable. Specifically, we define $x_{s}=f(x)$, where f is a function of a kind of linear transformation. The characteristic of the MBN signal is positively correlated with its feature uncertainty. The greater the instability of MBN signal is, the greater the feature uncertainty. We know that different confidence intervals in the feature joint probability distribution refer to different value ranges of feature variables. The larger the confidence interval is, the greater the feature uncertainty. Considering the three confidence intervals (68.2%, 95.4% and 99.7%) shown in Figure 5, we would like to construct three different multiple sample spaces from a sampler that provides samples from the multivariate Gaussian distribution of the features.

According to the reparameterization method, we assume ζ∼N(0,1) and $x_{s}=kA\circ \zeta +\mu$, where ∘ is pointwise multiplication, $\Sigma =AA^{T}$ is a Gaussian distribution with mean vector μ and covariance matrix Σ, and k is the coefficient that is set to increase the diversity of the sampling transformation. One convenient choice for A is to use the Cholesky decomposition of the covariance matrix $\Sigma =AA^{T}$. We should know that computing the Cholesky factorization of a matrix is symmetric and positive definite and that the covariance matrices calculated in our problem just meet this requirement. Three types of reconstructed sample space with different value ranges can be parameterized as

(5) $$X_{s}=\{\begin{array}{c} \mu \pm \Sigma^{1/2}\zeta ,\;[\mu -\sigma ,\mu +\sigma ]\\ \mu \pm 2\Sigma^{1/2}\zeta ,\;[\mu -2\sigma ,\mu +2\sigma ]\\ \mu \pm 3\Sigma^{1/2}\zeta ,\;[\mu -3\sigma ,\mu +3\sigma ] \end{array}$$

### 3.2. Model Uncertainty Analysis of Bayesian Priors

We specify the conditional probability distribution of the output given the input for a particular parameter setting. The Gaussian likelihood is then defined as

(6) $$p(y|x,\theta )=N(y|x^{T}\theta ,\sigma^{2}I)$$

Previously, we looked at linear regression models where we estimated the model parameters θ by means of maximum likelihood estimation (MLE:$\theta_{ML}=\arg\max_{\theta }p(Y|X,\theta )$) or maximum a posteriori estimation (MAP:$\theta_{MAP}=\arg\max_{\theta }p(\theta |X,Y)$). In practice, both MLE and MAP use a point estimate from which single specific parameter values are calculated and easily lead to overfitting. Bayesian linear regression allows us to reason about model parameters, that is, to place an a posteriori probability distribution over plausible parameter settings when making predictions. This means that we do not fit any specific parameters but introduce uncertainty in linear model parameters, which is achieved by approximating the corresponding distributions over all the parameters (prior and posterior distributions are considered). The probability relationship between the priors and the posterior probability distribution can be established by Bayesian theory:

(7) $$p(\theta |X,Y)=\frac{p(Y|X,\theta )p(\theta )}{p(Y|X)}$$

Although the prior and its corresponding posterior do not necessarily obey the same distribution in the actual situation, in order to facilitate calculations in the experiment, we only consider the general ideal situation. That is, we assume that the prior probability and posterior probability follow the same form of distribution with different parameters. We place a Gaussian prior distribution over the model parameters. Then, we consider the priors (prior distributions) and likelihood:

(8) $$p(\theta )=N(\theta |\mu_{0},\Sigma_{0})$$

(9) $$p(Y|X,\theta )=N(y|x^{T}\theta ,\sigma^{2}I)$$

According to Equation (7), we know that the posterior is proportional to the product of the prior and the likelihood, so if both the likelihood and prior distributions are Gaussian, then the posterior can also be modeled by a Gaussian distribution. We assume the posterior of model parameters

(10) $$p(\theta |D)=N(\theta |\mu_{p},\Sigma_{p})$$

where $\mu_{p}$ and $\Sigma_{p}$ can be parameterized over the parameters of priors and likelihood based on Bayesian theory (7). In some detail,

(11) $$p(\theta |X,Y)=N(\theta |\Sigma_{p}(\Sigma_{0}^{-1}\mu_{0}+\sigma^{-2}x^{T}y),\;\;{\left(\Sigma_{0}^{-1}+\sigma^{-2}x^{T}x\right)}^{-1})$$

From the above formula, we know that $\Sigma_{p}$ is a function of $\Sigma_{0}$, and $\mu_{p}$ is a function of $\mu_{0}$ and $\Sigma_{0}$. The selection of priors tends to be subjectively biased. In general, enough sample data can largely eliminate this prior bias. Any priors will yield roughly the same results; this is also known as the prior robustness of Bayesian learning. However, when there is a lack of sample data, as in our experiment, the limitations of the sample information and prior bias will lead to a large number of blind searches, causing low efficiency and precision of the posterior [30,31]. The fitting degree of the posterior parameters to the data depends largely on prior selection. That is, the closer the prior is to the true distribution, the faster and more accurately the posterior distributions can be found. From another perspective, we ignore the restrictive effect of the data and make the priors and the posterior as consistent as possible. Then, the result will be more ideal. On this basis, we compute the parameters $\mu_{0}$ and $\Sigma_{0}$ of the prior distributions on the regression coefficients θ to minimize the Kullback–Leibler divergence with the functional Bayesian posterior:

(12) $$p^{*}(\theta )=\underset{\mu_{0},\Sigma_{0}}{\arg\min}KL(p(\theta )||p(\theta |D)$$

Let

(13) $$\begin{array}{l} L(\theta )=KL(p(\theta )||p(\theta |D)\\ \;=\frac{1}{2}(tr(\Sigma_{p}^{-1}\Sigma_{0})+{\left(\mu_{1}-\mu_{0}\right)}^{T}\Sigma_{p}^{-1}(\mu_{1}-\mu_{0})-k+\log(\frac{\det\Sigma_{p}}{\det\Sigma_{0}})) \end{array}$$

where k is the dimensionality of the distribution. Then, we respectively calculate the gradient of L(θ) with respect to the mean $\mu_{0}$ and standard deviation $\sigma_{0}$ ($\sigma_{0}$ is the root of elements on the diagonal of covariance matrix $\Sigma_{0}$). The prior parameters are updated as $\mu_{0}=\mu_{0}-\alpha \Delta_{\mu_{0}}$ and $\sigma_{0}=\sigma_{0}-\alpha \Delta_{\sigma_{0}}$. Once the priors are obtained, the posterior is updated based on Equation (11). Then, we iterate to solve the more ideal posterior probability distribution. The iterative process uses the posterior probability parameters calculated at each step to readjust (or replace) the prior, and then the corresponding posterior is calculated again. If the effect of the second posterior is better than that of the first through some measurement parameters or methods, then this process continues until the newly obtained posterior probability no longer changes (converges). Otherwise, the iteration is terminated. Finally, based on the above reasoning, we obtain the predictive distribution of $y_{*}$ at an arbitrary test input $x_{*}$ as

(14) $$p(y_{*}|X,Y,x_{*})=N(y_{*}|x_{*}^{T}\mu_{p},x_{*}^{T}\Sigma_{p}x_{*}+\sigma^{2})$$

## 4. Experimental Results and Discussion

Based on a review of other studies on MBN signals, a commonly used preselected feature set (including energy, rms, mean, entropy, skewness, and the peak of the power spectral density) is used for our experiment, in which the three types of features are included. We use a sliding window with a length of a signal period to split continuous MBN signals and directly calculate the features of the single wave packet. The uncertainty in the MBN signals is modeled based on their multiple wave packets. The experiments in this paper were verified by 10-fold cross-validation (CV). To compare the prediction results of different methods, the prediction uncertainties are measured (one for mean and one for variance). The regression evaluation index root mean square error (RMSE) and r-square ($R^{2}$) are calculated as well.

### 4.1. Interval Sensitivity Analysis Based on Disorder Sampling

As described in Section 3.1, we statistically analyze the feature matrices corresponding to different wave packets measured under the same fatigue state based on Equations (3) and (4). This is used to create their associated probability distributions. Then, a set of probability distributions $T_{M\times K}$ is defined to characterize the uncertainty in features, in which the elements are represented in the form of Equation (2). M denotes the measuring moments m=1,2,⋯,M. Then, we set up three different sensitivity intervals corresponding to the three predictive confidence intervals 68.2%, 95.4%, and 99.7% of the standard normal distribution to reconstruct the sample space.

Based on Equation (5), three sets of sample space consistent with the probability distribution T are reconstructed, where the value of Gaussian noise ζ is randomly sampled from the standard normal distribution to simulate the stochastic condition of the MBN signal. The exact uncertainty interval conditions are listed in Table 3. The sensitivity interval ±∞ in Table 3 represents the original sample space, which is preserved to facilitate comparison with the reconstructed sample space. The lager the sensitivity interval is, the more scattered the feature distribution.

The interval sensitivity analysis of the predictions is performed by exploring the change rule of prediction uncertainty, as shown in Table 4. Table 4 lists the statistical information of the predicted mean $E(R_{i})=\frac{1}{S}\sum_{j=1}^{S}R_{i,j}\;\;i=1,2,\cdots ,m$ and standard error $V(R_{i})=\sqrt{\frac{1}{S-1}\sum_{j=1}^{S}{\left(E(R_{i})-R_{i,j}\right)}^{2}}\;\;\;i=1,2,\cdots ,m$ for the prediction results of each fatigue loading time under the conditions of using the original sample space and the reconstructed sample space as model inputs. $R_{i}$ represents the fatigue state response of predictions, and S is the total number of test data points for each fatigue state.

**Figure 6:**
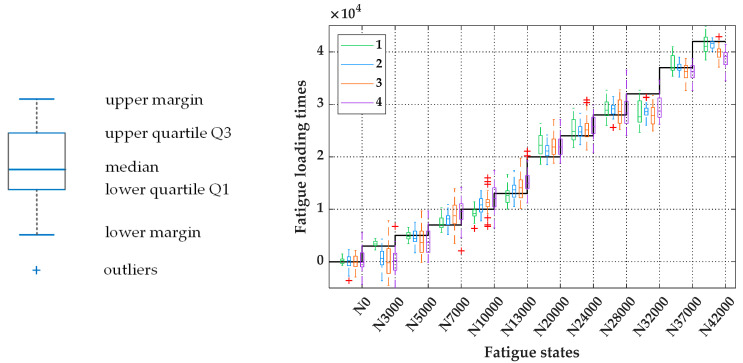
Comparison of prediction uncertainty using the four different sample spaces.

Figure 6 is a box plot corresponding to the statistical information in Table 4, where the black broken line describes the change in fatigue loading times corresponding to each fatigue state in the test dataset. The overall prediction accuracies, characterized by RMSE and $R^{2}$, are listed in Table 5. According to Table 4, Figure 6, and Table 5, it can be observed that the larger the sensitivity interval that is set, the greater the prediction uncertainty is. At the same time, the prediction accuracy is significantly reduced, indicating that the observation uncertainty will directly affect the uncertainty in and accuracy of the predictions. In addition, by comparing the prediction results from the original sample space (sensitivity interval: ±∞) and the reconstructed sample space with a sensitivity interval of 68.2% (sensitivity interval: ±σ), we found that their prediction accuracy and uncertainty under different fatigue states are somewhat similar. To some extent, we have successfully characterized the uncertainty conditions of feature distributions in the original sample space. On the basis of adding random Gaussian noise, the uncertainty of the MBN signals can be highly restored within the [μ−σ,  μ+σ] confidence interval of the feature distributions.

### 4.2. Feature Reparameterization Based on Ordered Sampling

According to Section 4.1, the stochastic condition of MBN signal is defined by analyzing feature uncertainty using interval sensitivity analysis. However, our idea is not limited to this approach. Our purpose is to improve the robustness of features through feature reconstruction on the basis of retaining feature uncertainty. We know that the stochastic quality of the MBN signal is intuitively presented in the disordered form of signal wave packets measured under the same fatigue state. Therefore, we must perform the noise quantization on these feature differences, making the different features extracted from the same wave packet have a consistent law to follow based on the random sampling within a confidence interval centered on the mean. Here, quantization refers to applying the same Gaussian noise value $\zeta_{i}∼N(0,1),\;i=1,2,\cdots ,N$ to the feature reconstruction in each example of the sample space. All reconstruction sensitivity intervals are selected as ±σ based on the conclusion of Section 4.1. Following this idea, a more robust feature sample space is reconstructed.

**Figure 7:**
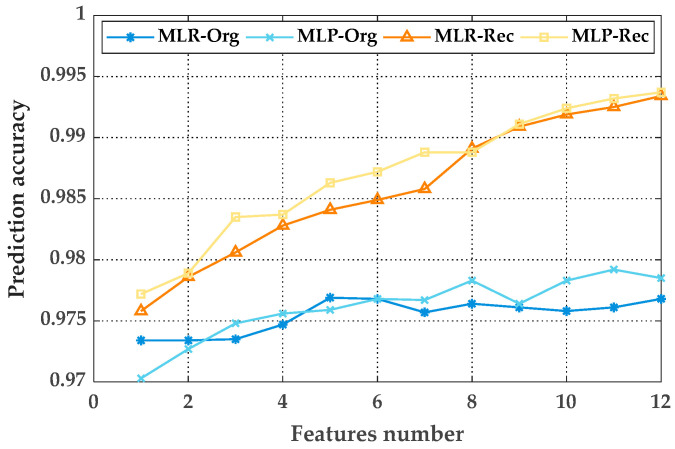
The prediction for various multiple linear regression (MLR) models.

To better compare the quality of features before and after reconstruction, the mapping between inputs and outputs is established by using two model types: multiple linear regression (MLR) and multilayer perceptron (MLP). The prediction accuracies of different numbers of features are shown in Figure 7. We found that no matter how many features are selected, the prediction results of the reconstructed features are superior to those of the original features. For the original features, the prediction accuracy improved slightly when the number of features increased, indicating that the feature dimension has little effect on the improvement of prediction results. However, for the reconstructed features, as the number of features increased, the prediction results improved significantly, and the prediction accuracy value was closer to 1. According to the analysis, the positive addiction effect is produced between features, and we infer that, as long as there are enough features that can be selected, the prediction accuracy can be infinitely close to 1.

**Figure 8:**
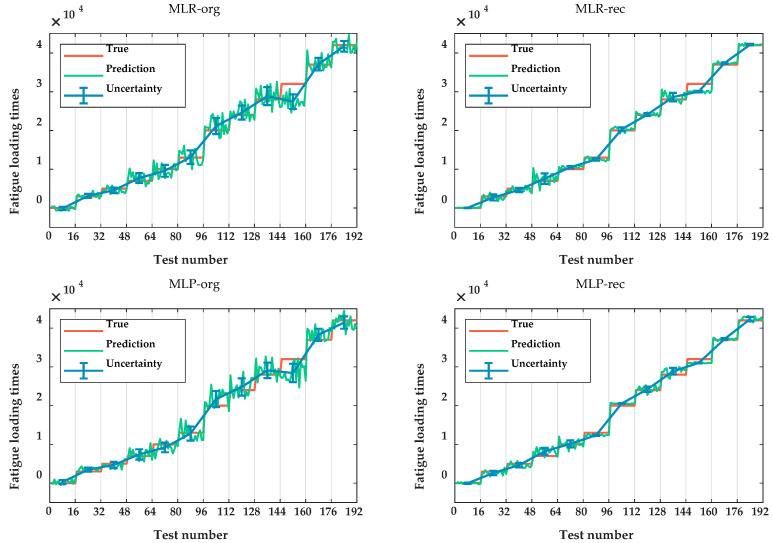
Prediction comparison of original and reconstructed feature sample spaces using MLR and MLP.

A prediction comparison using 12 features is presented in Table 6. Figure 8 shows detailed information about the prediction uncertainty of various fatigue states using MLR and MLP. It can be concluded that, no matter which model is chosen, the reconstructed features can greatly improve the prediction accuracy and reduce the output uncertainty compared with the original features. To some extent, the feature uncertainty analysis using the reparameterization sampling method with ordered random noise is beneficial for eliminating the influence of the stochastic characteristic of MBN signal. This provides clear distinctions between the reconstructed features associated with different fatigue states (labels in experiments) so that the models can more easily recognize different labels.

### 4.3. Developed Priors Setting for Model Uncertainty Measurement

Despite the popularity of the training methods of MLE and MAP, a lack of parameter uncertainty measures makes MLR prone to making overconfident predictions. In addition, regularization is always needed. Taking parameter uncertainties into account, the Bayesian linear regression (BLR) developed with MLR is applied, in which Bayesian estimation is used to estimate the probability distributions of regression coefficients. In BLR, priors (prior distributions) are of the highest significance and represent the knowledge accumulation of the researchers about the model parameters being estimated. Actually, the diagonal Gaussian distribution is typically used as the priors in the absence of prior knowledge, with the aim of relying on a good amount of data and multiple iterations to approximate a robust posterior. However, this greatly fails to make the prediction accuracy equivalent to that of MLE. A new method of prior setting is introduced in Section 3.2., and the effect will be verified and compared with that of MLE. Table 7 lists the posterior probability distributions trained by our method and the diagonal Gaussian distribution. The optimal parameters given by MLE are also listed.

**Figure 9:**
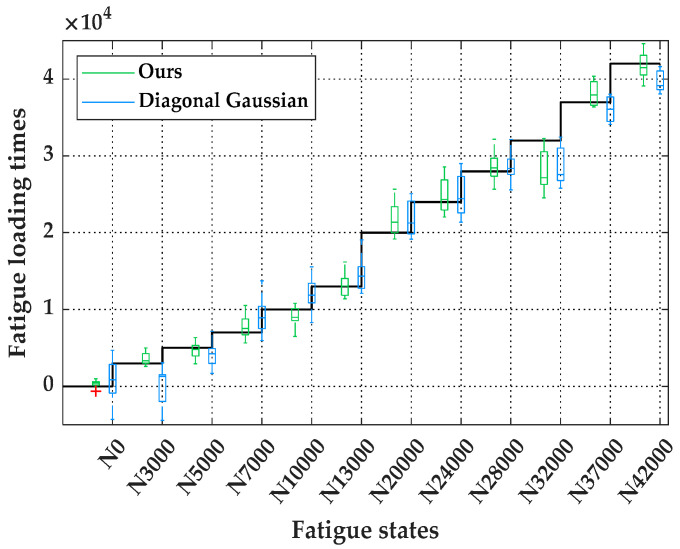
Comparison of prediction uncertainty using different priors setting.

Figure 9 shows the prediction uncertainty obtained when using the two types of priors. According to Table 6 and Figure 9, the prediction results of our proposed method for prior setting outperform the commonly used one. Meanwhile, the effect of our method is almost equivalent to that of MLE. This indicates that assigning a prior distribution reasonably is important for improving the efficiency and accuracy of model prediction and that our proposed method can enhance the precision of prior assignments without any informed prior information. Meanwhile, we found that, no matter which priors are used, the uncertainty of the obtained posterior parameters has little effect on the prediction results and can be ignored. This indicates that improvements in parameter uncertainties cannot be directly propagated to improve prediction uncertainty.

## 5. Conclusions

Understanding the stochastic characteristic of MBN signals is of great significance for improving the efficiency and accuracy of material property analysis. In this paper, a series of uncertainty and sensitivity analyses using Bayesian statistics is applied to model uncertainty in MBN signal features, model parameters, and predictions characterized as multivariate probability distributions. Feature uncertainty usually manifests as signal instability and is often difficult to suppress or evaluate. Model uncertainty is caused by unstable parameter tuning in the process of training. In addition, the uncertainty of the output is measured correspondingly as the input or model parameters change under different probability uncertainties. The main contributions of this paper include the reconstruction of the original feature space by reparameterization sampling. Through experimental analysis, we finally determined that the original feature space can be better restored by modeling within the probability confidence interval of [−σ,+σ] and adding random noise that obeys the standard normal distribution. The probability confidence interval is thus used as the measurement index of the stochastic quality of MBN signal. On this basis of reconstruction, interval [−σ,+σ] is maintained, and a more robust feature space can be obtained by adding the uniform quantization Gaussian noise to each example in the feature space, making the features extracted from the same wave packet have the same random distribution characteristics. The results proved that the performance of reconstructed features is much better than original features, and a good linear superposition effect is generated between the features. With the increase of feature dimensions, the prediction accuracy has been significantly improved. Moreover, to measure the uncertainties of the model parameters given that both the prior knowledge and data volume are limited, we proposed a more useful method for incorporating informative prior into training using the method of approximating Kullback–Leibler divergence between the prior distribution and posterior distribution. The criteria for selecting the optimal priors were chosen with the goal of maintaining a consistent prior and posterior. The results showed that our method is superior to the commonly used diagonal Gaussian prior and is comparable to MLE.

The analysis of MBN signal uncertainty proposed in this paper is not limited to the current experimental background. Rather, this analysis is expected to be further verified and expanded in other application scenarios, such as hardness and stress testing and material characteristic curve calibration.

## Figures and Tables

**Table 1:** The chemical composition of steel 20R (: %).

C	Si	Mn	P	S	Ni	Cr	Cu
0.17~0.23	0.17~0.37	0.35~0.65	≤0.035	≤0.035	≤0.03	≤0.25	≤0.25

**Table 2:** Parameters table of signal acquisition.

Parameters	Value
Excitation frequency	9 Hz/16 Hz/35 Hz
Output voltage of signal generator	0.5 V
Sampling frequency	1 MHz
Barkhausen wave packet number	150
Power magnification	10
MBN signal magnification	20
Bandpass filtering	5 kHz–50 kHz

**Table 3:** The setting of uncertainty intervals used in the experiment.

Serial Num	1	2	3	4
**Sensitivity interval**	±∞	±σ	±2σ	±3σ

**Table 4:** The uncertainty in the predictions.

Serial Num	1	2	3	4
**0**	268.47 ± 0.52$\;\times \;10^{3}$	46.29 ± 1.42$\;\times \;10^{3}$	−202.78 ± 1.67$\;\times \;10^{3}$	−169.63 ± 2.64$\;\times \;10^{3}$
**3000**	3483.88 ± 0.67$\;\times \;10^{3}$	2774.34 ± 1.98$\;\times \;10^{3}$	1308.89 ± 3.14$\;\times \;10^{3}$	1177.93 ± 3.13$\;\times \;10^{3}$
**5000**	4680.88 ± 0.81$\;\times \;10^{3}$	4663.44 ± 1.43$\;\times \;10^{3}$	3891.78 ± 2.45$\;\times \;10^{3}$	3999.97 ± 2.73$\;\times \;10^{3}$
**7000**	7414.28 ± 1.33$\;\times \;10^{3}$	8032.62 ± 1.27$\;\times \;10^{3}$	8959.23 ± 2.37$\;\times \;10^{3}$	9244.01 ± 2.62$\;\times \;10^{3}$
**10,000**	9197.50 ± 1.23$\;\times \;10^{3}$	10,749.56 ± 1.62$\;\times \;10^{3}$	11,264.93 ± 1.99$\;\times \;10^{3}$	11,997.91 ± 2.77$\;\times \;10^{3}$
**13,000**	12,793.82 ± 1.81$\;\times \;10^{3}$	13,537.09 ± 1.55$\;\times \;10^{3}$	13,978.91 ± 2.24$\;\times \;10^{3}$	15,265.55 ± 2.46$\;\times \;10^{3}$
**20,000**	21,699.65 ± 2.07$\;\times \;10^{3}$	21,135.74 ± 1.28$\;\times \;10^{3}$	22,116.94 ± 1.96$\;\times \;10^{3}$	22,448.67 ± 3.16$\;\times \;10^{3}$
**24,000**	24,810.07 ± 2.21$\;\times \;10^{3}$	24,876.67 ± 1.54$\;\times \;10^{3}$	25,253.09 ± 2.42$\;\times \;10^{3}$	25,771.84 ± 2.46$\;\times \;10^{3}$
**28,000**	29,094.94 ± 1.99$\;\times \;10^{3}$	29,142.15 ± 1.30$\;\times \;10^{3}$	28,612.62 ± 2.25$\;\times \;10^{3}$	28,719.35 ± 3.03$\;\times \;10^{3}$
**32,000**	28,419.50 ± 2.38$\;\times \;10^{3}$	28,615.83 ± 1.21$\;\times \;10^{3}$	27,961.96 ± 1.90$\;\times \;10^{3}$	29,384.89 ± 2.32$\;\times \;10^{3}$
**37,000**	38,271.63 ± 1.57$\;\times \;10^{3}$	36,976.86 ± 0.86$\;\times \;10^{3}$	36,156.01 ± 1.55$\;\times \;10^{3}$	34,802.78 ± 1.63$\;\times \;10^{3}$
**42,000**	41,429.98 ± 1.60$\;\times \;10^{3}$	41,416.01 ± 0.78$\;\times \;10^{3}$	39,830.73 ± 1.25$\;\times \;10^{3}$	38,786.25 ± 1.59$\;\times \;10^{3}$

**Table 5:** Comparison of prediction accuracy.

Serial Num	1	2	3	4
RMSE (Root Mean Square Error)	2010.91	2012.71	2962.47	3378.40
$R^{2}$ (R-square)	0.9766	0.9765	0.9476	0.9310

**Table 6:** Prediction accuracy of using different training data.

Method Num	Method	Features	RMSE	R^2^
1	Multiple Linear Regression, MLR (Maximum Likelihood Estimation, MLE)	$X/X_{s}$	2110.91/1126.33	0.9766/0.9934
2	Multilayer Perceptron, MLP (Back Propagation, BP)	$X/X_{s}$	2040.65/1070.12	0.9771/0.9937

**Table 7:** Parameter posterior calculation using different training methods.

Priors Condition		Diagonal Gaussian	Ours	MLE
**Posterior**	$x_{1}$	9.97 × 10^4^ ± 5.63 × 10^−3^	2.217 × 10^3^ ± 4.12 × 10^4^	2.684 × 10^3^
	$x_{en}$	−8.88 × 10^2^ ± 7.25 × 10^−6^	5.102 × 10^3^ ± 9.37 × 10^−7^	4.932 × 10^3^
	$x_{rm}$	−6.22 × 10^5^ ± 2.75	−9.476 × 10^6^ ± 2.00	−8.997 × 10^6^
	$x_{ma}$	−7.15 × 10^3^ ± 9.06 × 10^−2^	2.854 × 10^4^ ± 4.67 × 10^−4^	2.794 × 10^4^
	$x_{etr}$	−1.02 × 10^6^ ± 4.28	1.941 × 10^6^ ± 2.22	1.516 × 10^6^
	$x_{sk}$	5.16 × 10^2^ ± 3.95 × 10^−4^	−6.507 × 10^3^ ± 2.17 × 10^−5^	−6.198 × 10^3^
	$x_{psd}$	−4.08 × 10^4^ ± 5.93 × 10^−3^	5.317 × 10^4^ ± 4.43 × 10^−4^	4.858 × 10^4^
**Estimation**	RMSE	2795.27	2014.41	2110.91
	$R^{2}$	0.9524	0.9765	0.9766
